# Supervised Machine Learning Algorithms Can Classify Open-Text Feedback of Doctor Performance With Human-Level Accuracy

**DOI:** 10.2196/jmir.6533

**Published:** 2017-03-15

**Authors:** Chris Gibbons, Suzanne Richards, Jose Maria Valderas, John Campbell

**Affiliations:** ^1^ Centre for Health Services Research University of Cambridge Cambridge United Kingdom; ^2^ The Psychometrics Centre University of Cambridge Cambridge United Kingdom; ^3^ Leeds Institute for Health Sciences University of Leeds Leeds United Kingdom; ^4^ Primary Care Research Group University of Exeter Exeter United Kingdom

**Keywords:** machine learning, surveys and questionnaires, feedback, data mining, work performance

## Abstract

**Background:**

Machine learning techniques may be an effective and efficient way to classify open-text reports on doctor’s activity for the purposes of quality assurance, safety, and continuing professional development.

**Objective:**

The objective of the study was to evaluate the accuracy of machine learning algorithms trained to classify open-text reports of doctor performance and to assess the potential for classifications to identify significant differences in doctors’ professional performance in the United Kingdom.

**Methods:**

We used 1636 open-text comments (34,283 words) relating to the performance of 548 doctors collected from a survey of clinicians’ colleagues using the General Medical Council Colleague Questionnaire (GMC-CQ). We coded 77.75% (1272/1636) of the comments into 5 global themes (innovation, interpersonal skills, popularity, professionalism, and respect) using a qualitative framework. We trained 8 machine learning algorithms to classify comments and assessed their performance using several training samples. We evaluated doctor performance using the GMC-CQ and compared scores between doctors with different classifications using *t* tests.

**Results:**

Individual algorithm performance was high (range *F* score=.68 to .83). Interrater agreement between the algorithms and the human coder was highest for codes relating to “popular” (recall=.97), “innovator” (recall=.98), and “respected” (recall=.87) codes and was lower for the “interpersonal” (recall=.80) and “professional” (recall=.82) codes. A 10-fold cross-validation demonstrated similar performance in each analysis. When combined together into an ensemble of multiple algorithms, mean human-computer interrater agreement was .88. Comments that were classified as “respected,” “professional,” and “interpersonal” related to higher doctor scores on the GMC-CQ compared with comments that were not classified (*P*<.05). Scores did not vary between doctors who were rated as popular or innovative and those who were not rated at all (*P*>.05).

**Conclusions:**

Machine learning algorithms can classify open-text feedback of doctor performance into multiple themes derived by human raters with high performance. Colleague open-text comments that signal respect, professionalism, and being interpersonal may be key indicators of doctor’s performance.

## Introduction

Multisource “360-degree” feedback is increasingly used across business and health sectors to give workers insights into their performance and to identify areas in which improvements may be made. Such feedback often includes different reporting modalities that most commonly take the form of validated questionnaires or open-text comments. In the United Kingdom, large-scale national surveys include open-text feedback, such as the Friends and Family Test, the Inpatient Survey, and the Cancer Patient Experience Survey.

The complexity of open-text information means that, unlike the scores from validated patient-reported experiences and outcome measures, the words cannot simply be “added up” to create insight and meaning. As such, the task of making sense of such data has historically been completed manually by skilled qualitative analysts.

As the volume of text increases, qualitative data can quickly become difficult to manage and draw insights from. Coding and interpreting large bodies of qualitative information received from open-text comments collected is labor-intensive and is at risk of bias if multiple raters use subtly different coding heuristics. Where human raters systematically analyze qualitative data, there remain issues with both time and financial constraints of doing so, as well as potential challenges in ensuring intercoder consistency [1].

The term *machine learning* refers to the application of a growing number of algorithms that are able to complete diverse computational tasks, including mastering complex computer games [2], understanding the meaning of sentences [3], and successfully predicting psychological profiles from the Internet behavior [4,5].

Although machine learning appears to be eminently suitable for the task of classifying open-text data from national surveys, its potential is largely untested in the context of comments made by medical professionals about doctors’ performance. Classification algorithms have been previously applied to patient comments about the experience of living beyond cancer [6], clinical incident reports [7,8], and sentiment analysis of digital footprints including Twitter and online blogs [9,10].

While algorithms have demonstrated excellent performance in diverse tasks, there is no evidence specifically relating to their ability to classify comments about doctors made by their colleagues as part of a formal evaluation. Although doctors’ performance might be best assessed by fellow professionals who know them very well, positive reporting bias in open-text reports may occlude differences in performance [11,12]. The challenge therefore is to classify differences in text that is often positively worded and to use these classifications to signal differences in doctors’ performance.

The objective of this study was to train and evaluate an ensemble of machine learning algorithms to accurately classify open-text reports of doctors, which are known to be positively biased, and to assess the potential for theory-based classifications in open text to signal differences in doctors’ professional performance in the United Kingdom.

## Methods

### Sample

We collected data from all non–training-grade doctors from 11 sites in England and Wales between March 2008 and January 2011. We recruited doctors from 4 acute hospital trusts, an anesthetics department, 1 mental health trust, 4 primary care organizations, and 1 independent (non–National Health Service) health care organization. We provided all doctors with detailed information regarding the study before they consented to take part in it; they were told they could withdraw at any point without justification. Detailed description of this sample is reported elsewhere [7,8].

Doctors were asked to suggest up to 20 colleagues (half of whom were to be medically qualified) who could provide multisource “360-degree” feedback regarding their professional performance.

Multisource feedback was elicited using the General Medical Council Colleague Questionnaire (GMC-CQ), a reliable measure of doctor performance that is validated for use in the United Kingdom [13]. The GMC-CQ contains 18 items assessing diverse aspects of doctor performance and a section for entering open-text feedback.

### Text Categorization

Qualitative analysts inductively coded the open-text feedback from the GMC-CQ into 5 themes relating to (1) innovation and openness to change (59/1636 comments, 3.6%); (2) interpersonal skills and caring (432/1636 comments, 26.4%); (3) popularity (131/1636 comments, 8%); (4) professionalism (701/1636 comments, 42.8%); and (5) respect or esteem in which the doctor was held (346/1636 comments, 21.1%) [12]. We refer to these categories throughout the rest of the paper as innovator, interpersonal skills, popularity, professionalism, and respect. Classification of a comment into more than one theme was possible. Of the 1636 reports, 1211 (74%) were classified as belonging to at least one of these categories. Similarly, classification of doctors into more than one category was possible, and 648 (28.8%) reports were classified into one or more of the 5 categories; as such, there were 2858 human-labeled comments in the entire corpus.

The number of comments in each category, the distribution of words, and statistical comparison of the word length are provided in Table 1. Significant analysis of variance (ANOVA; with post hoc Tukey test) results indicate that the number of words in texts that were granted the label of “innovator” was significantly greater than all other categories, whereas comments which received a label or “respected” or no label at all were significantly shorter.

**Table 1:** Number of comments, distribution of words, and statistical comparison for each of the 5 categories.

Categories	Reports in category	Length of report, mean (SD)	ANOVA^a^*P* value
Innovator	59	41.99 (30.84)	<.001
Interpersonal	432	23.87 (16.39)	.99
Popular	131	25.49 (16.74)	.97
Professional	701	24.46 (17.34)	.91
Respected	346	20.69 (19.13)	.03
More than 1 category	1189	21.63 (16.76)	.56
No categories	425	19.54 (13.62)	<.001

^a^ANOVA: analysis of variance; conducted with post hoc Tukey tests.

The qualitative researchers followed Holsti’s approach [14]. Using rigorous data coding and verification procedures, which included double coding and independent verification within a qualitative framework [12], the resultant data were coded in such a way as to support quantitative data analysis.

Comments were generally, though not always, positive. In our sample, 91.5% (1497/1636) of all comments were positive, 5.93% (97/1636) of the comments were mixed, containing both a positive and a negative statement about the doctor, and the remaining 2.57% (42/1636) of comments were either neutral or negative (see Table 2). Prior publications relating to this dataset give further information on the process of ascertaining the polarity of comments [12].

### Assessment of Machine Learning Algorithm Performance

**Figure 1:**
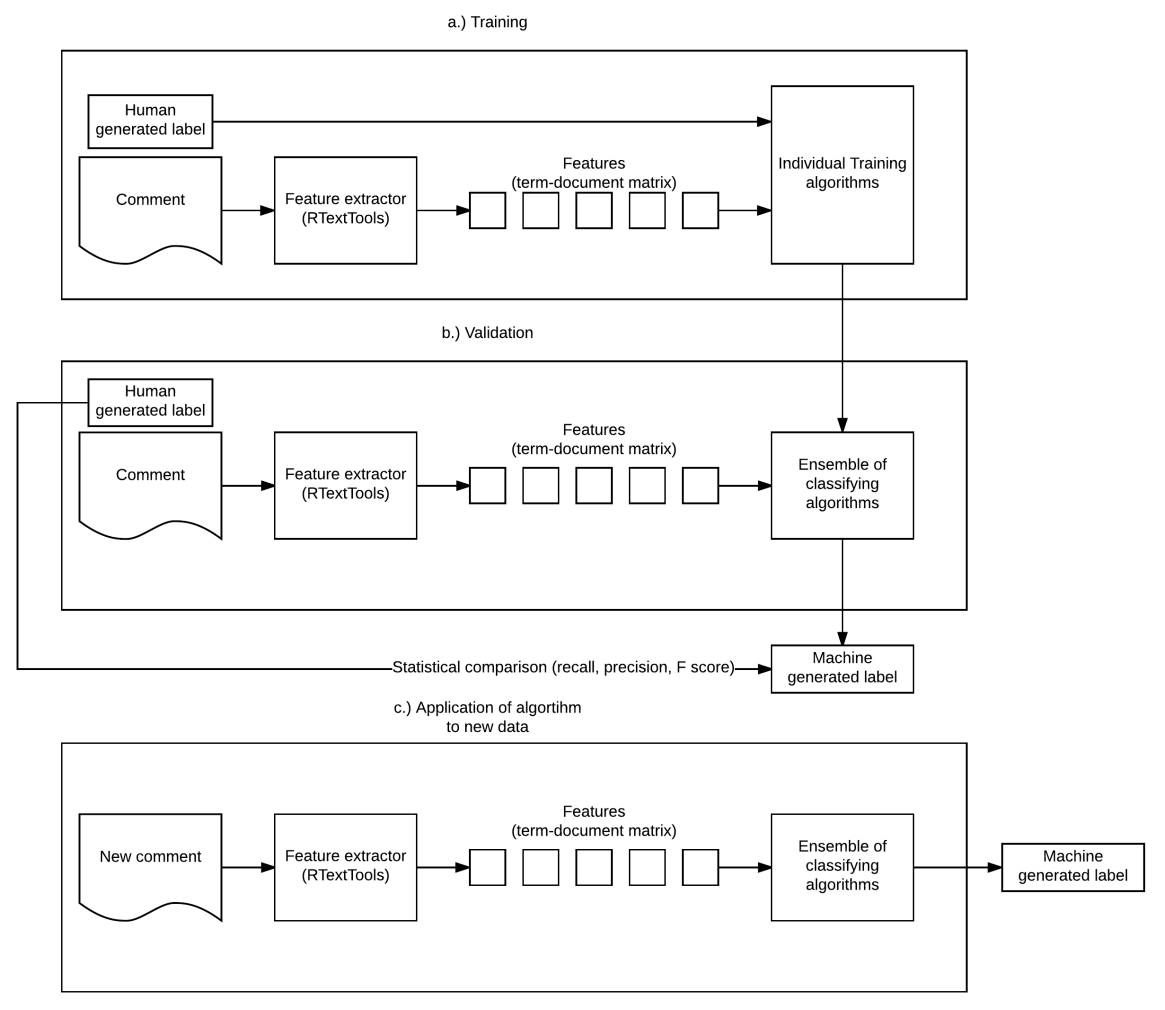
Flow diagram of the stages “training,” “validation,” and “application to new data.”.

The process of training, validating, and deploying the algorithms is illustrated in Figure 1.

**Table 2:** Example quotes from each category

Theme	Comment
Innovator	“It is clear from the advice he gives that he is aware of [the] current good practice, is highly motivated, very practical and very much a team player. His advice, when working with consultant colleagues was respected, and he recognized where practice/primary care limitations were and yet looked for opportunities for change and improvement.”
	“She has an admirable level of commitment and enthusiasm for her patients and her work. She has been instrumental in promoting change and improvement in her department. She is a great asset to the department and the hospital.”
Interpersonal	“She is a very good, committed colleague always keen to improve, very liked by her patients and highly valued by all who work with her.”
	“Very approachable and professional.”
Popular	“Excellent well liked and easy working colleague.”
	“Very popular doctor. Works to high standards.”
Professional	“I find this doctor to be very efficient, caring, honest and very professional.”
	“I find that he very easy and helpful to work with, he always has time for patients and staff.”
Respected	“A first class colleague.”
	“Pleasant and valued colleague.”
Not coded by qualitative rater (given label of 0)	“Supportive colleague, excellent time management skills.”
	“I think I have a good working relationship with this doctor. I have been impressed with his openness to Psychological work with his patients and his support for my work. In my opinion he gives thorough consideration to his diagnosis.”

### Feature Selection

The first step within each stage is the identification of features within the comments. The features used in this study were identified and stored using a term-document matrix that describes the frequency of terms that appear in each of the comments. The term-document matrix uses a bag-of-words structure that counts the number of terms in each comment and does not consider the order in which the words appear. Term-document matrices are a simple way to represent text data that are computationally straightforward. The matrix comprised unweighted words and was cleaned by stemming, removing numbers, and removing sparse terms (where a certain word was only used in fewer than 0.02% of cases) [15]. Sparse-term removal reduced the number of terms from 1737 to 616. The final term-document matrix contained a lexicon of 616 unique words (columns) for 1636 comments (rows). The matrix density was 5.8%.

The term frequencies for each comment were therefore used as features that the algorithms used to classify the text. An example of a term-document matrix is provided in Table 3. For each of the 5 categories, texts with a human classification in that category were labeled with a 1 and those without were labeled with a 0.

**Table 3:** An example term-document matrix for 3 texts.

Texts	Terms											
	a	and	colleague	doctor	great	is	patients	respected	this	troublesome	well	with
Text 1^a^	1	0	1	0	1	0	0	0	0	0	0	0
Text 2^b^	1	0	1	0	0	0	0	0	0	1	0	0
Text 3^c^	0	1	0	1	1	1	1	1	1	0	1	1

^a^Text 1: “A great colleague.”

^b^Text 2: “A troublesome colleague.”

^c^Text 3: “This doctor is well respected, and great with patients.”

Once the features have been extracted, they are used to “train” the algorithms to describe the relationship between the features and the classification.

In the validation stage the classifications given by the ensemble to new data are compared with the classifications made by the human qualitative analysts. If the results of the validation stage are acceptable, the algorithms can be exported and used to classify new data independently of the dataset that was used to train and validate the models.

The steps of these stages are given in greater detail in Figure 1.

### Algorithms

“RTextTools” brings together other packages that contain different machine learning algorithms and provides a system by which the performance of each algorithm can be assessed both individually and as a collected ensemble of different methods that are combined to maximize performance in the training dataset. We included all of the available algorithms within RTextTools apart from the neural network, which did not converge in pilot assessments. The algorithms were support vector machine (SVM) using the radial basis function kernel with the penalty parameter of error term set to 1 and a gamma parameter set to 1/number of features [16], scaled linear discriminant analysis (SLDA) with eigenvalue threshold set to ≥1, bootstrapped boosting (bagging) with 25 bootstrap replications [17], boosting [18], random classification and regression forests with 500 trees [19], classification and regression tree [20], maximum entropy without regularization [21], and generalized linear models with L1 (lasso) penalized regularization (GLM/LASSO) [22].

A review and summary of supervised machine learning algorithms can be found elsewhere [23].

### Training and Validation

Algorithms were trained with a corpus of 1000 randomly selected precoded comments (see Figure 1, part “a”) and validated on the remaining 636 comments (see Figure 1, part “b”).

We assessed algorithm performance using statistics of (1) recall (analogous to sensitivity)—what proportion of cases in a class are correctly assigned to the class; (2) precision (analogous to specificity)—how often a case that is predicted to belong to a class does belong to that class; and (3) *F* score, which is a combination of both recall and precision where 1 represents the best performance and 0 the worst performance [24]. To maximize performance, algorithms are combined into a consensus “ensemble” consisting of multiple algorithms. The consensus ensemble is a collection of algorithms that make the same prediction concerning the class of a text in the training dataset. We included the group of algorithms that had full agreement on every document in the dataset in the training sample. Classification was performed using majority voting between the algorithms in the ensemble.

When assessed as an ensemble of multiple algorithms working together, recall is evaluated alongside coverage (the proportion of cases within the dataset to which the recall value applies) [21]. The *F* value is analogous to interrater reliability and, as such, we will accept agreements ≥.80 between the algorithms and the human codes as evidence that the algorithms can complete the categorization task with acceptable accuracy.

### n-Fold Validation

In addition to the standard assessment of algorithm performance using a validation dataset (636 comments), stability of algorithm performance across different data was also tested using an n-fold cross-validation. In the current analysis, a 10-fold validation was used in which 10 randomly selected samples of 1000 comments were selected from the dataset and validated using the remaining documents.

This analysis will indicate the robustness of the algorithms and their suitability for application to novel data and is preferable to split-half validation or bootstrapping [6,25].

### Sample Size Accuracy Trade-Off

As well as the precision of the algorithms it is important to assess their training efficiency (the relationship between performance and size of the training dataset) so that we might best understand how to apply these techniques in practice. We compared training efficiency performance using randomly selected training sets of 1000, 750, 500, 250, 100, and 50 to accurately classify randomly selected comments from a fixed-size validation set (636 cases).

### Assessment of Group Differences

To assess the ability of these categories to highlight differences in global performance, we investigated the differences in GMC-CQ scores for doctors whose comments were classified into at least 1 of 5 categories and those who were not placed in any category. We hypothesized that doctors who were placed into one or more categories would perform better than those doctors who were not classified into any of the categories.

We conducted this analysis using both the machine-coded dataset (the entire dataset was recoded by the algorithm blinded to the codes given by the human rater and the original human-rated dataset.

Questionnaire data were scored using the graded response model [22]. All items fit the graded response model (chi-square interaction *P*>.01) and overall model fit was good (root mean square error of approximation =.048, comparative fit index =.97) [26,27]. The scale’s marginal reliability was .76. This analysis was conducted so that interval-scaled logit scores (theta) could be extracted from the model to use in the comparative analysis. This technique has been shown to increase sensitivity to detect change on questionnaire measures of quality of life [28]. Correlation between theta values and scale raw scores was .95. Further details on the process of item response theory scoring and analysis can be found elsewhere [27,29,30].

### Statistical Analysis

Computational text classification and statistical analysis were conducted within the R statistical programming environment (R Foundation) [31] using the “RTextTools” package for training the algorithms and the “base” package for conducting between-group comparisons. Figures were plotted using “ggplot2.”

### Ethical Approval

The study was originally considered by the Devon and Torbay NHS Research Ethics Committee but judged not to require a formal ethics submission. No subsequent ethical approval was sought for the secondary analyses on the anonymized datasets presented here.

## Results

### Assessment of Algorithm Performance

Table 4 presents the summary performance statistics for the algorithms and their individual *F* scores and the recall values for the ensemble of algorithms.

**Table 4:** Summary of algorithm and ensemble performance in the main analysis.

Model^a^	Metric	Innovator	Interpersonal	Popular	Professional	Respected	Average
Support vector machine	*F* score	.73	.69	.84	.79	.73	.76
Scaled linear discriminant analysis	*F* score	.77	.65	.88	.73	.77	.76
Boosting	*F* score	.75	.77	.81	.76	.75	.77
Bootstrap boosting	*F* score	.87	.85	.83	.80	.82	.83
Random forests	*F* score	.67	.59	.87	.78	.74	.75
Decision tree	*F* score	.80	.75	.88	.78	.80	.80
Generalized linear model	*F* score	.89	.82	.88	.81	.89	.85
Maximum entropy	*F* score	.70	.62	.73	.65	.70	.68
Final ensemble (3+ models with agreement for the entire dataset)	Recall with 100% agreement	.98	.80	.97	.82	.87	.89
10-Fold validation mean (range)	*F* score	.97 (.96-.98)	.80 (.74-.86)	.97 (.96-.98)	.79 (.75-.83)	.86 (.84-.89)	.88

^a^Training set size=1000; validation=636.

#### Innovator

The GLM/LASSO algorithm was the single highest-performing algorithm for correctly classifying the open-text comments into the “innovator” category. The ensemble of 3 algorithms (GLM/LASSO, bootstrapped boosting, and regression tree) has a 98% recall agreement with the human coder. The 10-fold validation indicated robust accuracy scores between .96 and .98 (mean .97). In the whole dataset, 48 comments (3.5%) were classified as an innovator by the algorithms.

#### Interpersonal

The bootstrapped boosting algorithm was the best-performing algorithm for categorizing open-text comments into the “interpersonal skills” category. The ensemble of 3 algorithms (boosting, GLM/LASSO, and bootstrapped boosting) demonstrated an 80% recall agreement with the human-coded dataset, the lowest performance for any of the classes. The 10-fold validation indicated similar performance in each fold and agreement values between .74 and .86 (mean .80). The algorithms classified 435 comments (28.4%) as “interpersonal.”

#### Popular

All algorithms performed exceptionally well in classifying open-text comments into the “popular” category, with *F* scores greater than .80 for all but maximum entropy (*F* score=.73). The ensemble performance (SLDA, decision tree, and GLM/LASSO ) was also excellent, with an interrater recall agreement of .97 (10-fold validation range .96-.98, mean .97). In total, 107 comments (8.3%) were placed in the “popular” category.

#### Professional

Similar performance was evident for many algorithms including SVM, random forests, and GLM/LASSO . Overall ensemble performance (GLM/LASSO, bootstrap boosting, and SVM) had an interrater recall of .82. The 10-fold validation suggested good agreement between the algorithm and the human analyst (mean .79, range .75-.83). The algorithms classified almost half of the comments in the whole dataset into the “professional” category (643 comments, 42.7%).

#### Respected

Once again, the GLM/LASSO algorithm showed the strongest single performance in the classification task for the “respected” category (*F* score=.89). The overall performance of the ensemble was very high, with .87 recall between the human coder and the 3-algorithm ensemble. The 10-fold validation demonstrated greater agreement between the human analyst and the algorithms (mean .86, range .84-.89). In the whole dataset, the ensemble classified 243 (16.6%) comments into the “respected” category.

#### Overall Performance

The GLM/LASSO algorithm was the strongest performing individual algorithm and the maximum entropy the worst. The overall average performance was nevertheless high (*F* score=.77). Average agreement between the human coder and the ensemble of algorithms was high (.89).

### n-Fold Validation of Ensemble Accuracy

Results for the 10-fold cross-validation were very similar for the final recall values for the individual samples. The n-fold result displayed a tight distribution over the 10 samples (Table 4), indicating that the ensemble performs robustly across different samples.

### Algorithm Performance With Differing Sample Sizes

**Figure 2:**
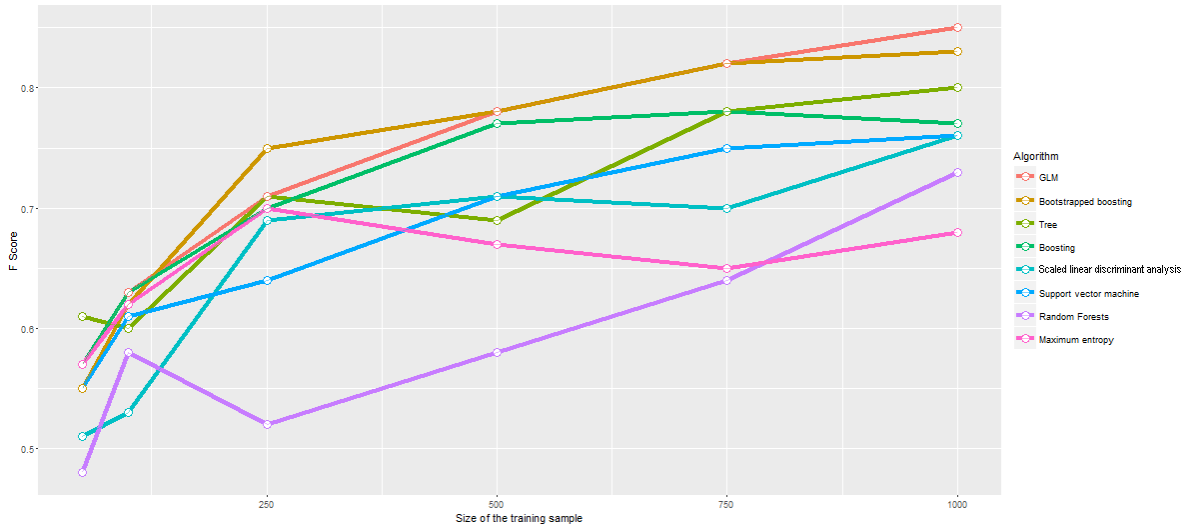
Algorithm performance with differing training sample sizes. Performance decreases as expected with smaller training corpora.

As the training sample size was reduced, the algorithms continued to perform well but fell sharply when the training dataset was reduced to fewer than 250 comments. Figure 2 shows the algorithm performance with different training sample sizes.

### Assessment of Group Differences

**Figure 3:**
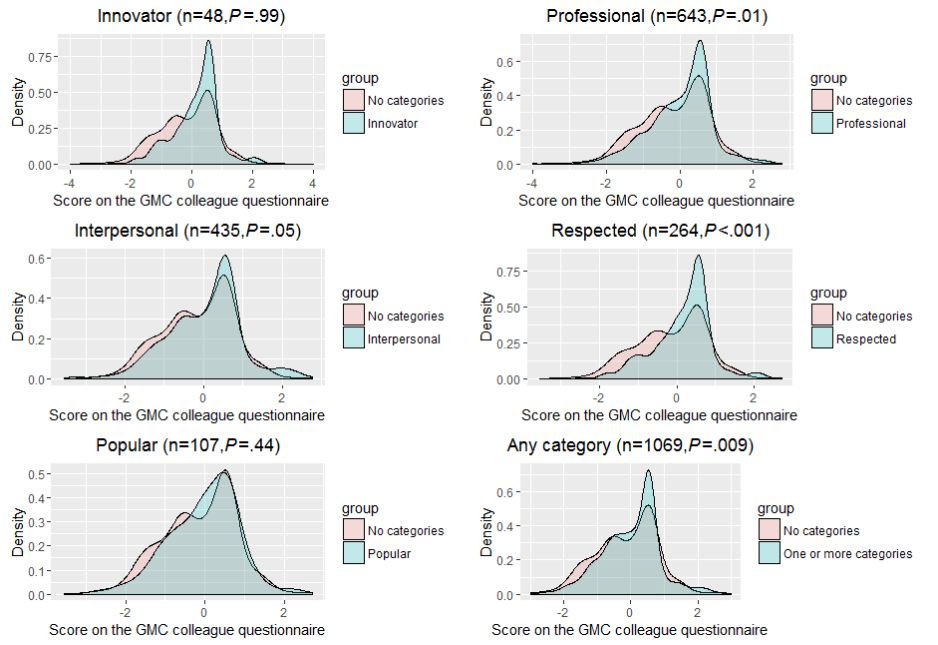
Comparison of General Medical Council Colleague Questionnaire (GMC-CQ) scores between doctors who were placed in 1 of the 5 categories versus those who were not (positive comments only). Significance (P) values for the t tests are shown to indicate the relationship between the 2 groups.

The *t* tests demonstrated a significant difference in the GMC-CQ scores between doctors who received comments that placed them into 1 of the 5 categories and those who did not (*t*_173.81_=0.77, *P*=.001). Although the results were significant, there was sizeable overlap in the distributions as shown in Figure 3, indicating that open-text classification alone was not sufficient to reliably distinguish between doctors’ performance. The largest difference in mean performance was between doctors who were classified as “respected” and those without a classification (*t*_629.17_=3.75, *P*<.001). There was no difference in mean performance scores between doctors who were classified as “popular” (*P*=.44) and those who were not. Similarly, being rated as “innovative” did not signal higher performance (*P*=.99), although the low numbers in the analysis suggest a lack of power to detect an effect (n=48). Table 5 presents the results of these analyses conducted with both the machine learning classifications (Table 5, “Panel A”) and the human classifications (Table 5, “Panel B”). The results are similar between the human-rated and machine-rated datasets, with stronger effect sizes being reported in the human-classified group.

**Table 5:** Comparison of means between doctors classified into a category and those who were unclassified.

Categories	Panel A^a^						Panel B^b^				
	Mean score (logits)	Reports in category	*t* test (vs no category rating)				Mean score (logits)	Reports in category	*t* test (vs no category rating)		
			*t*	df^c^	*P*				*t*	df	*P*
Innovator	0.00	48	0.00	55.74	.99		0.01	59	1.14	35.69	.26
Interpersonal	1.97	435	1.98	857.97	.04		0.07	432	2.97	346.63	<.01
Popular	−0.05	107	−0.88	176.42	.38		0.13	131	1.32	149.05	.19
Professional	−0.03	643	2.51	901.34	.01		0.1	701	3.47	286.99	<.001
Respected	0.15	243	3.75	629.17	<.001		0.44	346	5.58	300.13	<.001
More than 1 category	0.04	1081	0.77	173.81	.001		0.12	1189	3.81	239.8	<.001
No categories	−0.09	413	N/A^d^	N/A	N/A		−0.4	425	N/A	N/A	N/A

^a^Panel A: analysis using machine ensemble classifications on entire corpus.

^b^Panel B: analysis using human rater classifications on entire corpus.

^c^df: degrees of freedom.

^d^N/A: not applicable.

## Discussion

### Principal Findings

This study demonstrates the ability of machine learning algorithms to categorize qualitative data with high performance. The integration of such algorithms into data analysis tool kits for nationwide surveys may allow rich qualitative data to be analyzed without the resource burden associated with expert human ratings across an entire corpus [32].

We also demonstrate the ability of categories to highlight differences in overall doctor performance that were statistically significant. We hypothesized that doctors who were classified into 1 of the 5 categories would have higher scores on the GMC-CQ than those who were unclassified. We found partial support for this hypothesis: doctors who were classified as “respected,” “professional,” and “interpersonal” tended to outperform unclassified doctors, whereas no significant difference in performance was evident between doctors who were classified as “popular,” “innovative,” and those who were not classified into any of the 5 categories. However, the number of doctors who received a classification of “innovative” was low, which resulted in tests with low power and potential for type II error. Doctors with multiple ratings performed better than those without any ratings. Doctors who were classified as “respected” had the highest performance of each group in both the human-rated and machine-rated datasets.

These techniques have clear potential for developing actionable insights in diverse specialties: they have also been used to classify patient-derived open-text comments in national cancer surveys [6]. A key advantage of these techniques is the possibility of deploying trained algorithms to operate on data as they are being collected, allowing real-time feedback and insight from open-text data [33], which may be used to monitor performance, and possibly safety, in the future. It is important to remember that although machine learning algorithms can perform to a high level in prediction or classification tasks, some operate as “black box” and it is often difficult, or even impossible, to generate theory or convey insight into *how* the algorithms arrived at their final solutions.

We trained algorithms using precoded data and validated their performance on uncoded data, an example of “supervised” machine learning. We demonstrate strong performance using a relatively simple sparse “term-document matrix” method of identifying features in open text. The term-document matrix simply counts the instances of a word’s use within a comment and does not consider the order in which the words are presented. This approach has been used in similar studies published in the medical literature [6,8].

It is possible to extract features using different, more complex, methods. Feature extraction using *n*-grams offers a means to retain some of the context in which words are used. An *n*-gram tokenizes sequences (of length *n*) of words as features, which may provide better information than the simple word-count strategy utilized in term-document matrix. Similarly, dimension reduction or clustering techniques such as latent Dirichlet allocation or singular value decomposition may have been used to reduce the sparsity within the matrix. There are no simple guidelines suggesting the optimal matrix density for use in this context and the possible benefits of clustering and dimension reduction must be counterbalanced by the caveat that these techniques can reduce the interpretability and accuracy of predictions [4].

Although performance of these techniques is demonstrably high, further research may be warranted to explore the extent to which they can improve the accuracy of classification algorithms in this context and at what cost (eg, computational burden or interpretability).

Natural language processing algorithms and their related software, driven by market forces in the technology industry, are improving at a remarkable rate and, paradoxically given the economic motivation for their development, increasingly being distributed at no cost under open-source licenses. As the field develops, we may expect these sorts of algorithms to successfully classify more complex corpora and perhaps even identify important elements within open-text comments without task-specific training data, which is known as “unsupervised” machine learning.

### Limitations

This study has some limitations. The average performance of our algorithms is likely to be high in some instances given the low incidence of category. For example, algorithm performance was exceptionally high (recall=.97) for the “innovator” category, where only 4.2% of doctors were rated as innovative. In this instance, the low number of classifications in the population would have meant that a “dumb guess” that simply rated each doctor as “not innovative” would have demonstrated a 95.8% agreement rate. However, while algorithm performance was somewhat lower for categories with balanced distributions (eg, 46% of doctors were rated as professional), it was still acceptably high (recall=.82, 10-fold accuracy=.87).

Because of a somewhat small dataset, the trained ensemble was used to reclassify the whole corpora on which the algorithms were originally trained. It is likely that the performance of the algorithms will be higher when reclassifying the data they were originally trained on. The rationale for this decision was to maximize the number of classified categories and therefore maintain statistical power in the analyses. The results of these assessments were broadly the same as the results from the human-classified dataset, albeit the effect sizes were consistently smaller in the analyses using the machine-classified codes. This may be especially important as it appeared that the machine-labeled dataset was less sensitive to differences in doctor’s performance than the human-labeled dataset.

A further possible limitation of the dataset was the necessity to have a small training-set to validation-set ratio (3:2) to keep sufficient number of comments in the validation sample. While this advantaged the statistical analysis of difference in performance signaling for different categories, it may have hampered the performance of certain algorithms by not providing sufficient training data.

The significant positive skew in doctors’ ratings and the scarcity of comments that were outright negative meant that we were unable to conduct a sentiment analysis. We expect this to be the case in a population where most subjects are performing well, and this is probably representative of most datasets that collect open-text information on doctor performance. The content of uncategorized comments reveals a trend of doctors saying *something* positive about the colleagues if it did not relate to key elements of their medical practice (eg, “supportive colleague. Excellent time management skills”). The data used here were collected in relation to the high-stakes General Medical Council revalidation exam, which may introduce a barrier to honest reporting of negative aspects of a doctor’s practice. In addition to contextual factors, innovation surrounding the manner in which such comments are elicited, including less direct conversational techniques, may also reduce reporting biases.

Although algorithm performance was generally high for each of the individual machine learning techniques, it is apparent that the generalized linear model with lasso regularization had the highest performance for each of the classes. The precise reason for this improved performance is somewhat opaque, but the lasso regularization technique is especially suitable for classification problems using sparse matrices [34]. It is somewhat surprising that classification and regression trees outperformed the random forests; this may be attributed to the sparsity of the matrix and the low number of classifications made in some of the categories leading to high misclassification error in the random trees. Their performance may have been improved using a dimensional reduction technique such as singular value decomposition or latent Dirichlet allocation, which reduces sparsity in the matrix but which may also lead to a loss of information for other algorithms and uninterpretable results [35,36].

Similarly, it is not immediately clear as to why certain codes could be classified with greater accuracy than others. The differences in performance between classes may be explained by differences in the conceptual basis of each class; both humans and algorithms may find it easier to classify comments that reflect easily defined concepts such as being “popular” (the class for which algorithm performance was highest), rather than less well-defined concepts such as being “interpersonal” (the class for which algorithm performance was lowest) [37].

There may be an opportunity for similar techniques to be applied to patient experience data to build algorithms that can correctly classify and perhaps, using sentiment analysis, quantify open text in national-scale patient experience surveys and provide feedback that is more meaningful to both patients and practitioners. Computational analysis of open-text comments may be of greater usefulness when it is used to identify issues that were not previously envisaged.

### Conclusions

This study demonstrates excellent performance for an ensemble of machine learning algorithms tasked to classify open-text comments of doctors’ performance. These algorithms perform well, even where limited time and resources are available to code training datasets. We demonstrate that machine identification of qualitatively derived, theory-based open-text classifications can signpost significant differences in a doctor’s performance, even when comments are exclusively positive. These findings may inform future predictive models of performance and support real-time evaluation to improve quality and safety.

## Abbreviations

ANOVA: analysis of variance
GMC-CQ: General Medical Council Colleague Questionnaire
NIHR: National Institute for Health Research
SLDA: scaled linear discriminant analysis
SVM: support vector machine
